# Supplementary material for: Metabolic reprogramming during neuronal differentiation from aerobic glycolysis to neuronal oxidative phosphorylation
Source: eLife. 2016 Jun 10;5:e13374. doi: 10.7554/eLife.13374 (PMC4963198; doi:10.7554/eLife.13374)
Supplement: Supplementary file 1. — DOI: http://dx.doi.org/10.7554/eLife.13374.030 [file elife-13374-supp1.pdf]

TABLE S1 Real time PCR primers

Gene	Primer sequence
ACO2	AATGGATGTACTCGTTGGGC
ACO2	ACAGCCTACTGGTGACTCGG
ACO2	ATGGATGTACTCGTTGGGCT
ACO2	TTGTCAGTGCACAAAATGGC
ATP5A1	AAGACACGCCCAGTTTCTTC
ATP5A1	TTTGGGTTCATCTTTCATTGC
ATP5B	CAAGTCATCAGCAGGCACAT
ATP5B	GTGGGCTATCAGCCTACCCT
ATP5E	CTTCAGTGCATCTCTCACTGC
ATP5E	TACAGCATGGTGGCCTACTG
β-actin	AAACTGGAACGGTGAAGGTG
β-actin	AGAGAAGTGGGGTGGCTTTT
COX5B	AGTCGCCTGCTCTTCATCAG
COX5B	TGGCTTCAAGGTTACTTCGC
COX6A1	AGCCAGTTGGAAGTGGATTC
COX6A1	TGTACCTGAAGTCGCACCAC
CS	GGGCTGCAAGAACAAGACA
CS	CTCCCTTTCTTACCTCCCCA
CYC1	GGTCACTGGCACTCACAGC
CYC1	CCGCTACGGACACCTCAG
CYCS	TGCCTTTCTCAACATCACCC
CYCS	GGCGTGTCCTTGGACTTAGA
ENO1	GCCTCCTGCTCAAAGTCAAC
ENO1	AACGATGAGACACCATGACG
ERRγ	GCTGTTCTCCGCATCTATCC
ERRγ	TGAAATCACAAAGCGCAGAC
FH	CCTCATCTGCTGCCTTCATT
FH	GGAGGTGTGACAGAACGCAT
GAPDH	TGTTGCCATCAATGACCCCTT
GAPDH	CTCCACGACGTACTCAGCG
IDH2	AACCGTGACCAGACTGATGAC
IDH2	ATGGTGGCACACTTGACAGC
MDH1	GGTGCAGCCTTAGATAAATACGC
MDH1	AGTCAAGCAACTGAAGTTCTCC
NDUFA1	TTCTAGCAGGGGTAGATGGC
NDUFA1	GAGATAGGCGCATCTCTGGA
NDUFB2	GAACTCGCTCTGGAACACCT
NDUFB2	ACTGCTGGAGATGGTGGAGT
NRF1	CCACGTTACAGGGAGGTGAG
NRF1	TGTAGCTCCCTGCTGCATCT
OGDH	TTGGCTGGAAAACCCCAAAAG
OGDH	TGTGCTTCTACCAGGGACTGT
PGAM1	TCGCTCTCTTCTGCACTGAG
PGAM1	ACCTGGAGAACCGCTTCAG

PGC1α	CTGCTAGCAAGTTTGCCTCA
PGC1α	AGTGGTGCAGTGACCAATCA
PGK1	AGCAGAATTTGATGCTTGGG
PGK1	TCTAACAAGCTGACGCTGGA
PKM	TCACTATGGGGCTTCGACAT
PKM	ATAACCTTGAGGCTGAGGCA
SDHA	CGAACGTCTTCAGGTGCTTT
SDHA	AAGAACATCGGAACTGCGAC
SDHB	CACAGATGCCTTCTCTGCAT
SDHB	AAGGCTGGAGACAAACCTCA
Sucla2	TTGCTTCAGGAGACTCAGCA
Sucla2	GTGAGCGAAAATATCCCAGG
TFAM	TCCAGTTTTCCTTTACAGTCTTCAG
TFAM	CCAAAAAGACCTCGTTCAGC
TFB1M	CACGTCCACCTCTGGTTTG
TFB1M	TCGCCTCTCTGTTATGGCTC
TPI1	ATGGCTGAAGTCCAACGTCT
TPI1	AAGGAAGCCATCCACATCAG
UQCRC2	GTGGCATGTAAGAACCAGCA
UQCRC2	TCCAACACAGATGTCCAAGC
HK2	AAGCCCTTTCTCCATCTCCT
HK2	CTTCTTCACGGAGCTCAACC
LDHA	GCCAGAGACAATCTTTGGTG
LDHA	GGCCTGTGCCATCAGTATCT
LDHA (ChIP)	TCCTGACTCAGGCTCATGGC
LDHA (ChIP)	AGACAACCGACCGGCAGA
HK2 (Myc)	GCCCCGCAGGTAGTCAGG
HK2 (Myc)	GACCACGATTCTCTCCACG
HK2 (HIF1)	CACATTGTTGCATGAAACTCC
HK2 (HIF1)	GACCTCTCCGATTCACAGG
ChIP control	CAGAAGTTTTACGAAGACAATGG
ChIP control	ATGGTTACAGCGGCTAAGC
